# Neighbourhood composition dictates expression of soil legacy effects on plant growth

**DOI:** 10.1371/journal.pone.0342996

**Published:** 2026-02-17

**Authors:** Kenneth J. Oppon, Charlotte Brown, Isaac Peetoom Heida, James F. Cahill

**Affiliations:** Department of Biological Sciences, University of Alberta, Edmonton, Alberta, Canada; National Cheng Kung University, TAIWAN

## Abstract

Soil legacies, which reflect the influence of previous vegetation on soil biota and other properties, are increasingly recognized as drivers of current plant performance and community dynamics. Yet the expression of these legacies often depends on a focal plant’s immediate neighbours. We tested how soil biota from adjacent grassland and wolf-willow (*Elaeagnus commutata*) shrublands—representing a common shrub-encroachment transition in the northern prairies—affected the growth of *Agrostis scabra* when grown alone or with grass (*Festuca hallii*) or forb (*Geum triflorum*) neighbours. When grown alone, *A. scabra* responded differently across soil origins: grassland soil biota had a neutral effect, whereas wolf-willow strongly reduced growth. Neighbour presence modified these outcomes. In wolf-willow soils, the negative soil biota effect was no longer detectable with either neighbour individually, but re-emerged when both neighbours co-occurred, indicating non-additive interactions. In grassland soils, *G. triflorum* alone induced a negative effect that disappeared in the multi-neighbour treatment. Differences in soil chemistry, particularly the lower pH of wolf-willow soils, may have further shaped how microbial communities mediate plant responses. Overall, our findings demonstrate that soil biota effects on plant growth are not fixed by soil origin but are dictated by a plant’s neighbourhood composition, including neighbour identity, density, and their interaction with the abiotic context. Understanding these context-dependent soil legacies is essential for interpreting and predicting vegetation change and species coexistence across communities.

## Introduction

For decades, theories of plant community dynamics were dominated by abiotic explanations, attributing successional change primarily to competition for limiting resources and gradual shifts in soil physical properties [1–3]. More recently, changes to the soil biotic community have been recognized as an equally crucial driver of these dynamics [4–6]. Unlike many abiotic processes, biotic soil legacies, defined here as plant-mediated changes to soil microbial communities driven by prior occupancy, can develop rapidly and influence plant communities on much shorter timescales [7,8]. This perspective is particularly relevant in ecosystems composed of a mosaic of vegetation states, such as savannahs, where transitions between shrublands and grasslands may not result from changes to abiotic factors alone but are actively mediated or reinforced by the changes to soil biota [9,10].

The expression of these soil legacies, however, is highly context dependent. While the specific microbial assemblage that forms a soil legacy is determined by the plants that created it [11,12], its effect on new individuals depends on the traits of the responding plant [13], local abiotic conditions [14,15], and critically, the surrounding neighbourhood of plants whose roots overlap or share soil microbial communities [16,17]. Neighbouring plants can modify the influence of soil biota in multiple ways—by competing for benefits conferred by mutualists [18], amplifying pathogen impacts [19], promoting distinct soil organisms [20], or restricting their establishment altogether [21,22]. Through such mechanisms, neighbour identity and density, and more broadly the composition of the surrounding plant neighbourhood, can generate fine-scale heterogeneity in how soil legacies manifest within plant communities.

Despite growing recognition of this complexity [23], much of our understanding of soil legacy effects arise from simplified experimental systems, such as plants grown in monocultures or in isolation [24]. While foundational, these approaches often overlook the non-additive and synergistic interactions that emerge in diverse community settings [17,25]. Although several studies have examined soil legacies in more diverse or field-based contexts, explicit manipulations of neighbour identity, density, and community context remain rare [26]. Consequently, the role of soil biota as context-dependent mediators of plant interactions remains underexplored in realistic, multi-species settings [6,27].

Beyond within community variation, soil legacies can also differ across contrasting vegetation states. Comparing the soil biota associated with distinct community types can reveal how vegetation shifts alter the belowground communities and, in turn, alter plant diversity and composition [10,28,29]. In the Aspen Parkland of Alberta, for instance, shrub encroachment by the nitrogen fixing *Elaeagnus commutata* Bernh. ex Rydb (wolf-willow) marks a successional shift from grassland to shrub-dominated states. While such transitions alter the chemical soil environment [30] they may also reshape the soil biotic community. This reshaping, in turn, could influence plant diversity in shrub encroached areas compared to shrub free counterparts [31]. However, because local plant neighbourhoods can further modulate these biotic effects [32,33], the influence of soil legacies are unlikely to be uniform across neighbourhood contexts.

Here, we test how variation in a plant’s local community—through changes in neighbour identity and density—modifies the influence of soil biota on plant growth. Specifically, we compare soils from wolf-willow shrubland and wolf-willow-free grassland communities on plants grown alone, and with interspecific neighbours at two densities. This approach allows us to assess whether the influence of soil biota is a fixed property of community type or an emergent outcome shaped by local neighbourhood interactions.

## Methods

A three-month greenhouse experiment was conducted to test how soil biota from grassland and wolf-willow shrubland communities in the Aspen Parkland ecoregion of central Alberta influence plant growth. These soils were selected based on evidence that wolf-willow encroachment alters community composition beneath the shrub [30,31]. Wolf-willow is a dominant actinorhizal shrub capable of increasing nitrogen inputs, altering soil pH, resulting in distinct understory assemblages compared to adjacent wolf-willow-free grasslands [34,35]. Although such abiotic differences are well documented, the extent to which wolf-willow also modifies soil biota and their subsequent effects on plant growth remains unclear.

The initial experimental design was factorial: we used soils collected from two community types (grassland and wolf-willow) x from 20 field plots x 2 sterilization treatments (live vs sterile) x 4 replicates, for a total of 320 pots. Each pot was sown with a three-species mixture consisting of two grasses, *Agrostis scabra* (L.) and *Festuca hallii* (Vasey), and a forb, *Geum triflorum* (Pursh). Pots received three seeds per species that were surface sterilized prior to sowing (see below). These species were selected because they commonly co-occur in grassland communities at our study site (32) and showed consistent germination in preliminary trials using locally collected seed.

**Fig 1:**
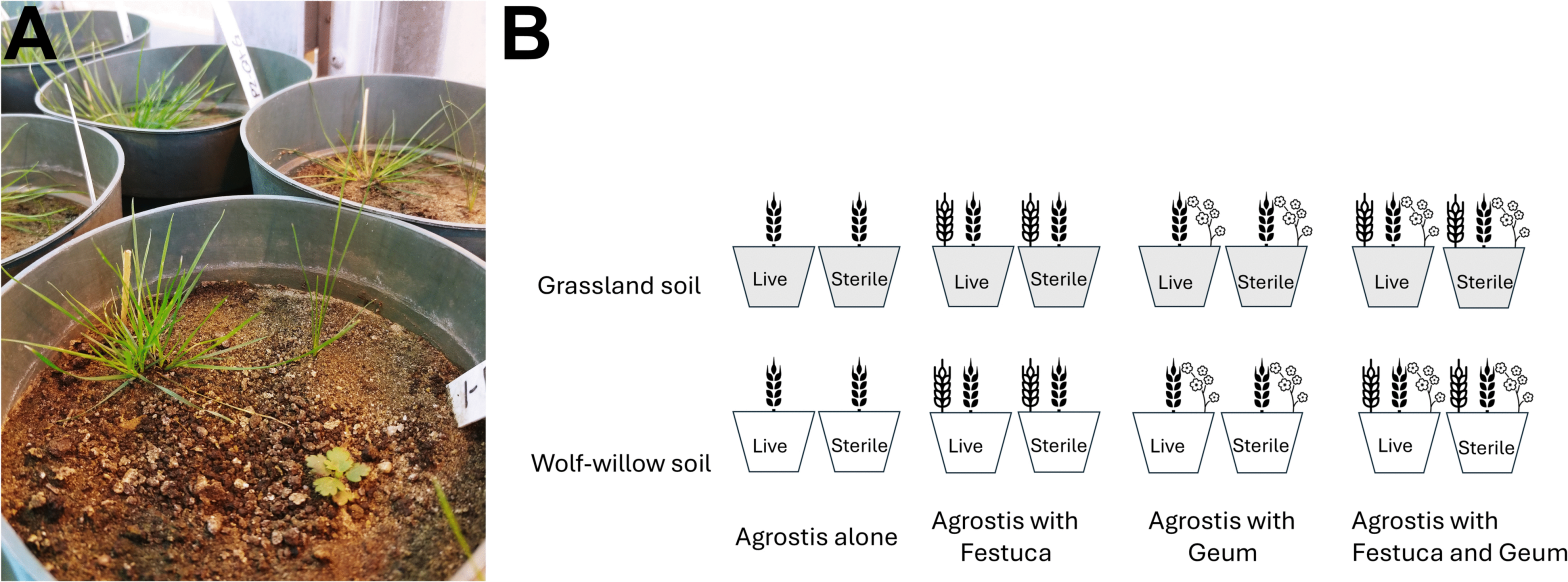
Setup of greenhouse experiment. **(A)** Photograph of pots representing a neighbour × soil origin × sterilization treatment. **(B)** Diagram illustrating the full set of neighbour, soil origin, and sterilization treatments.

However, variable germination of *F. hallii* and *G. triflorum* altered the final design, as *A. scabra* germinated most reliably (306 of the 320 pots). We therefore focused on *A. scabra* as the focal species to test how soil biota influenced its growth under four neighbour contexts: (1) *A. scabra* grown alone, (2) with *G. triflorum* as a neighbour, (3) with *F. hallii* as a neighbour, (4) or with both species together (Fig 1). Each species was represented by a single individual per pot, with excess seedlings removed shortly after germination. Although replication among neighbour treatments was unbalanced (Table 1), this structure allowed us to assess whether different neighbour scenarios modified the effect of soil biota on *A. scabra* growth.

**Table 1:** Shoot biomass (±SEM) of Agrostis scabra plants grown in four neighbour treatments across different soil origin and sterilization treatments. A priori comparisons were performed among measurements on individuals in adjacent rows and shaded in the same way. Boldface denotes significant differences based on planned pairwise comparisons of model estimates, with alpha set at 0.05. Treatments are illustrated in Fig 1.

Neighbour treatment	Soil origin and sterilization treatment	n	Shoot biomass (g)	p-value
Agrostis alone	Grassland live	9	0.682 ± 0.062	0.1141
	Grassland sterile	8	0.548 ± 0.039	
Agrostis with Festuca	Grassland live	30	0.600 ± 0.037	0.7121
	Grassland sterile	32	0.577 ± 0.032	
Agrostis with Geum	Grassland live	13	0.624 ± 0.059	**0.0529**
	Grassland sterile	4	0.816 ± 0.077	
Agrostis with Festuca and Geum	Grassland live	26	0.545 ± 0.027	0.5292
	Grassland sterile	32	0.584 ± 0.028	
Agrostis alone	Wolf-willow live	8	0.509 ± 0.060	**0.0288**
	Wolf-willow sterile	3	0.763 ± 0.044	
Agrostis with Festuca	Wolf-willow live	33	0.544 ± 0.033	0.1613
	Wolf-willow sterile	42	0.602 ± 0.029	
Agrostis with Geum	Wolf-willow live	8	0.528 ± 0.054	0.9795
	Wolf-willow sterile	8	0.531 ± 0.057	
Agrostis with Festuca and Geum	Wolf-willow live	25	0.526 ± 0.030	**0.0181**
	Wolf-willow sterile	24	0.646 ± 0.041	

### Field-soil origin and collection

Field soils were collected in September 2019 at the Roy Berg Kinsella Research Ranch (Kinsella, Alberta), a savannah type landscape with interspersed shrub and forest patches within predominantly herbaceous grasslands. Because this property is entirely owned and managed by the University of Alberta, and the study did not involve protected species or restricted areas, no external permits were required for site access or sample collection. At each of 10 locations within a 50- ha area, paired 3 x 3m plots established in a previous study were sampled [30]: one within a wolf-willow stand (mean density = 2.91 m ⁻ ²) and one in an adjacent grassland area (<1% wolf-willow cover) within 10 m.

Each plot was subdivided into four 1.5 x 1.5m quadrants. Within each quadrant, four soil cores (5 cm diameter × 20 cm depth) were collected 0.5 m inward from the corners, approximately 1m apart. Soils from the two upper quadrants and lower quadrants were pooled separately, producing two homogenized soil samples per plot. In total, there were 40 unique soils (10 locations × 2 community plots × 2 samples per plot). Soil samples were refrigerated at 4°C for four days before use.

### Greenhouse experiment

Seeds were collected from multiple locations within the study site over the two growing seasons prior to the experiment. All seeds were surface sterilized in a 5% bleach solution for two minutes then rinsed three times with Milli-Q water.

Pots (15 cm diameter x 14.5 cm depth) were filled with 1L of soil. Soils were a 9:1 ratio of sterilized background soil with live or sterilized field soil as inoculum. Background and inoculum soils were thoroughly homogenized within pots to ensure even distribution of microbial communities. This ratio minimized abiotic differences while maintaining representative microbial effects [36]. The background soil used is a 3:1 ratio of sand and topsoil (upper 20 cm; obtained from Canar Rock Products Ltd. in Edmonton AB, Canada). Sterilization of field and background soils was achieved by autoclaving for 3 hours at 121°C. Although we acknowledge that autoclaving may not eliminate all microbial propagules, this approach is has been used to substantially reduce viable soil biota and create a functionally sterile control for other plant–soil interaction experiments [37,38].

Three seeds of each species were sown into each pot across our four soil treatments: live grassland, sterile grassland, live wolf-willow, and sterile wolf-willow. Seeds were positioned approximately 2 cm inward from the pot edge and spaced approximately 2 cm apart. After germination, seedlings were thinned to one individual per species per pot. Pots were distributed across four spatial blocks, with positions randomized within each block. Plants were grown in the University of Alberta’s Biotron greenhouse, and pots were hand-watered from above every 1–3 days, adjusting the amount of water to maintain soils in a moist but not saturated condition. After three months, shoots of each species were clipped at the surface, dried at 60°C for 48 hours, and then weighed.

### Statistical analyses

All analyses were performed with R statistical software [39]. The data were visually inspected to confirm normality and homoscedasticity.

Effects of soil biota on *A. scabra* shoot biomass were tested using a linear mixed-effects model (function ‘lmer’ from the lme4 package v. 1.1.29, [40]) and evaluated with an ANOVA. Fixed factors included soil community origin (grassland, wolf-willow), soil sterilization (live, sterile), and neighbour treatment (alone, *F. hallii*, *G. triflorum*, both). Block was included as a random factor. Field plot origin explained negligible variance (SD = 0.00) and was excluded.

Potential outliers were assessed using a t-test on studentized residuals (function ‘outlierTest’ from the car package [41]). One observation was removed due to excessive influence (Bonferroni-adjusted p-value <0.01). Planned contrasts comparing live vs sterile soils within each neighbour treatment were conducted using the pairs function in *emmeans* [42]. Because these contrasts were predefined by the experimental design, no multiple comparison correction were applied.

## Results

**Fig 2:**
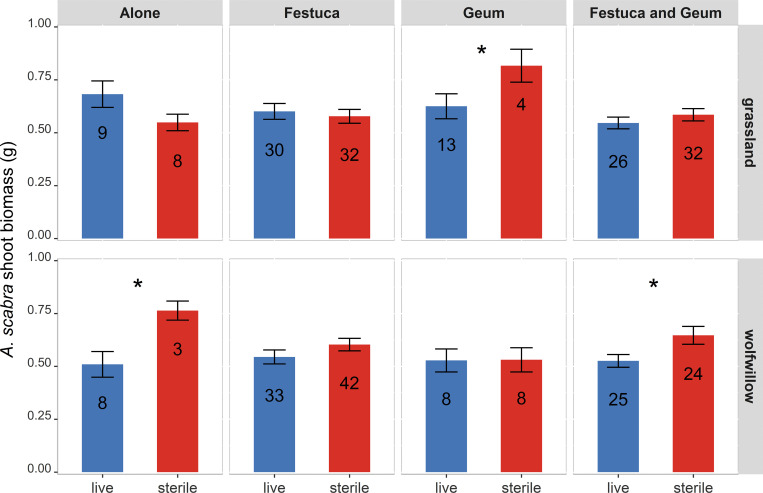
Mean (± SEM) shoot biomass of *Agrostis scabra* across neighbour treatments in live vs. sterile soils from two communities. Columns show neighbour identity; rows show soil community origin. Asterisks indicate significant live–sterile differences within neighbour treatments (α = 0.05).

There was a significant three-way interaction between soil sterilization, soil community origin, and neighbour treatment on *A. scabra* shoot production (*F*_*3,287.6*_ = 2.90, *p* = 0.03; Table 2). Because this interaction conditions all lower-order effects, we focus our interpretation on this, as the main effects and two-way interactions cannot be meaningfully interpreted in isolation. The result indicates that the effect of soil sterilization on shoot production depended jointly on soil origin and neighbour identity (Fig 2).

**Table 2:** ANOVA table for the effects of neighbour, community soil origin and sterilization treatments on Agrostis scabra shoot production.

Source of Variation	Shoot biomass			
	DF_num_	DF _den_	*F*-value	*P*-value
Neighbour	3	287.58	0.451	0.716
Community origin	1	286.03	0.732	0.392
Sterilization	1	286.37	4.782	**0.029**
Neighbour x Community origin	3	287.61	1.706	0.165
Neighbour x Sterilization	3	287.22	0.651	0.582
Community origin x Sterilization	1	286.39	3.736	**0.054**
Neighbour x Community origin x Sterilization	3	287.66	2.936	**0.033**

DF_num_ = Numerator degrees of freedom.

DF_den_ = Kenward-Roger adjusted denominator degrees of freedom.

In grassland soils, sterilization tended to reduce shoot production when *A. scabra* was grown alone (−20%), although this difference was not statistically significant (*p* = 0.11). When *G. triflorum* was present, sterilization significantly increased shoot production (+30%; *p* = 0.05), whereas no significant sterilization effects were detected when *A. scabra* grew with *F. hallii* or with both neighbours simultaneously (*p* > 0.1).

In wolf-willow soils, sterilization significantly increased shoot production when *A. scabra* was grown alone (+49%; *p* = 0.02). This positive effect of sterilization disappeared when either neighbour species was present individually (*p* > 0.1). However, when both neighbours were present simultaneously, sterilization again significantly increased shoot production (+20%; *p* = 0.01), suggesting that the joint presence of the two neighbours reinstated biotic constraints that were absent under single-neighbour conditions.

## Discussion

We found that the net effect of soil biota on the grass *A. scabra* was not fixed but varied with its local neighbourhood context. When grown alone, soils from grassland and wolf-willow communities produced divergent biotic effects on *A. scabra* shoot production: in grassland soils the effect of the biota was positive but not statistically significant, consistent with findings that early successional soils often exert neutral to positive effects on heterospecifics [8,43], whereas wolf-willow soil biota strongly limited growth. This contrast suggests that soil biota associated with wolf-willow communities impose stronger negative legacy effects on *A. scabra* consistent with broader evidence that plant performance differs across habitats due to variation in soil microbial community effects [6,24]. Our finding provides the first evidence that such biotic effects may contribute to performance differences between grassland and wolf-willow communities in the Aspen Parkland.

Crucially, these baseline soil legacy effects were not static. Neighbourhood structure altered both the direction and or magnitude of the soil biota effects, demonstrating that the expression of soil legacies is strongly context-dependent. Taken together, our results highlight that the influence of soil biota on plant growth is not fixed but emerges from the interaction between soil origin and a plant’s local neighbourhood [26,44]. This points to a need to move beyond a simplified pairwise perspectives and instead evaluate plant-soil interactions in their full community context.

In grassland soils, neighbour identity was the primary modifier of soil legacies. When *G. triflorum* was present, the effect of soil biota on *A. scabra* shifted from neutral to negative, as sterilization increased shoot production only in this neighbour context. This pattern indicates that *G. triflorum* altered the expression of soil biota effects rather than introducing new deleterious microbes. Similar findings in other systems show that specific neighbours can reshape rhizosphere conditions or filter soil microbial communities in ways that modify soil-legacy outcomes [22,45,46].

Functional differences between forbs and graminoids likely contribute to these neighbour-dependent outcomes, particularly through their distinct impacts through soil inputs. Forbs and graminoids often differ in root exudate quality and litter inputs into soils [47,48]. For instance, Steltzer and Bowman [49] demonstrated that *Geum rossii* (a congener of *G. triflorum*) modifies soil carbon and nitrogen dynamics differently than co-occurring grasses, potentially creating soil conditions that select for distinct microbial communities. Such species-specific root activity can rapidly select for distinct microbial communities even over short timescales [48].

Furthermore, our finding challenges the assumption that phylogenetic relatedness reliably predicts soil legacy effects. Ecological theory often predicts that closely related species should amplify soil effects through accumulation of shared host pathogens or mutualists [23,50]. However, in our case, the phylogenetically similar neighbour did not strongly influence the outcome. Rather, it was the phylogenetically distant forb, *G. triflorum*, that shifted the outcome from neutral to strongly negative in grassland soils. This aligns with recent arguments that relatedness is often a poor predictor of biotic soil effects because traits driving microbial associations do not strictly track phylogeny [51,52]. Specifically, this suggests that the forb neighbour promotes a distinct microbial assemblage that acts antagonistically toward *A. scabra*. *G. triflorum* likely recruits or tolerates microbial taxa that it can withstand, but which prove deleterious to *A. scabra*. By driving such shifts in the rhizosphere composition, local plant associations determine which microbial taxa become most ecologically influential [20,21].

In wolf-willow soils, however, the modifier of soil legacy effects shifted from neighbour identity to neighbourhood composition. When *A. scabra* grew alone, live soil biota had a clear negative effect on shoot production. Yet, in contrast to the identity-specific effects observed in grasslands, the presence of *either* neighbour individually nullified the negative soil biota effect*.* Strikingly, when both neighbours were present simultaneously, the negative soil-biota effect was again expressed, although at a reduced magnitude. This re-emergence of the negative effect suggests that increasing neighbourhood complexity—and the associated increase in plant density—alters how existing soil legacies are expressed [17,53–55].

Mechanistically, these non-additive outcomes likely arise because the rhizosphere that forms when species co-occur can differ markedly from those maintained by each species alone [48,56]. Rather than simply summing the distinct soil inputs described above, the combined presence of graminoids and forbs creates a unique rhizosphere environment. We propose that while each neighbour individually modifies the soil environment against harmful biota for *A. scabra in* wolf-willow soil, their combined interaction shifts rhizosphere conditions in a way that allows deleterious microbial components to reestablish influence [57,58]. These effects may be particularly pronounced when functionally distinct groups co-occur, as the interaction between differing belowground strategies can unpredictably shift microbial dominance [14,59,60]. Together, these results suggest that soil legacy effects emerge through neighbour-mediated changes in the expression of soil biota, rather than as predictable, additive extensions of pairwise plant–soil interactions [6].

An important context for understanding the net biotic effects we observed is the underlying soil chemistry of our study system. Previous studies have attributed performance differences between plants in grasslands and wolf-willow soils to variation in soil chemistry [34], specifically due to the nitrogen-fixing capacity of wolf-willow plants [35]. However, recent work at our study site found minimal differences in nutrient availability, including nitrogen, [30], but did report consistently lower pH in wolf-willow soils. Soil acidity can strongly shape microbial community composition and function [61–63], potentially favouring taxa less beneficial—or even antagonistic—to *A. scabra*. This chemical context helps explain the baseline differences we observed: the acidic stress of wolf-willow soils may promote a baseline community of antagonistic microbes (detectable when *A. scabra* grows alone), whereas grassland soils require the specific influence of *G. triflorum* to induce a similar negative effect. Essentially, the microbes that become influential only in the presence of *G. triflorum* in grassland soils may be naturally dominant under the more acidic conditions of wolf-willow soils, thereby driving the strong negative legacy observed in that that environment.

While these results reveal strong context-dependence in how plants experience soil legacies, several limitations should be acknowledged. Without direct characterization of the soil microbial communities or data on root colonization, the specific agents and mechanisms driving the observed interactions remain unresolved. Because we measured only aboveground responses, key belowground processes—such as root biomass allocation or mycorrhizal colonization—may have contributed to these patterns but were not captured [64,65]. Moreover, by focusing on a single focal species and a limited set of neighbour combinations, our ability to generalize to the broader community is restricted. Greenhouse conditions also constrain interpretation, as soil legacies and neighbour effects may shift under the variable and heterogeneous conditions of field environments [25,66]. Future studies that pair microbial sequencing with root-level analyses could identify the mutualists and antagonists mediating these soil legacies [67] while reciprocal field experiments would test whether the patterns observed here persist under natural environmental variability. Extending these approaches across multiple focal species and more diverse neighbour assemblages would clarify whether the context-dependence observed here is a general feature of soil legacies or a species-specific outcome.

## Conclusion

Together, these results offer a coherent picture of how local neighbourhoods and community legacies jointly shape plant performance. Our findings underscore that a plant’s immediate community context plays a crucial role in determining how it experiences biotic soil legacies. These effects were not fixed by the soil community's origin alone but shifted with neighbour identity, neighbourhood complexity, and through non-additive interactions among neighbours.

Across communities, *A. scabra* faced disadvantage through different pathways: In wolf-willow soils, negative soil-biota legacies strongly suppressed growth when plants grew alone, but the expression of this negative effect changed when neighbours were present individually and reappeared when neighbours co-occurred. In grassland soils, generally neutral legacies became negatively expressed only in the presence of particular neighbours such as *G. triflorum*. This contrast illustrates that soil legacies and neighbour effects interact in distinct ways across environments, producing outcomes that cannot be predicted from community type or soil origin alone.

In essence, soil-legacy effects emerge from fine-scale biotic interactions embedded within local neighbourhoods rather than from static community-level properties. In grassland-shrubland mosaics such as those represented in our study system, where community boundaries and species can be diffuse, this context dependence suggests that vegetation dynamics are driven by more than just soil conditioning. Specifically, the strong negative legacies observed in wolf-willow soils suggest that soil biota act as a barrier to the establishment of *A. scabra* (and potentially similar species), effectively filtering which species can colonize shrub patches. However, our finding that neighbours can mask these effects implies that this biotic resistance is not absolute. Consequently, the ability of *A. scabra* (and other similar species) to persist in or recolonize shrub-dominated soils may depend less on its intrinsic tolerance to shrub legacies and more on the buffering capacity provided by specific neighbour associations.
